# An Advanced Numerical Trajectory Model Tracks a Corn Earworm Moth Migration Event in Texas, USA

**DOI:** 10.3390/insects9030115

**Published:** 2018-09-05

**Authors:** Qiu-Lin Wu, Gao Hu, John K. Westbrook, Gregory A. Sword, Bao-Ping Zhai

**Affiliations:** 1 Department of Entomology, Nanjing Agricultural University, Nanjing 210095, China; wuqiulin89@126.com (Q.-L.W.); hugao@njau.edu.cn (G.H.); 2 Department of Entomology, Texas A&M University, College Station, TX 77843-2475, USA; 3 Insect Control and Cotton Disease Research Unit, US Department of Agriculture, Agricultural Research Service, College Station, TX 77845, USA; John.Westbrook@ARS.USDA.GOV

**Keywords:** numerical simulation, migration, Weather Research and Forecasting (WRF) model, three-dimensional trajectory analysis program, valuation, *Helicoverpa zea*

## Abstract

Many methods for trajectory simulation, such as Hybrid Single-Particle Lagrangian Integrated Trajectory (HYSPLIT), have been developed over the past several decades and contributed greatly to our knowledge in insect migratory movement. To improve the accuracy of trajectory simulation, we developed a new numerical trajectory model, in which the self-powered flight behaviors of insects are considered and trajectory calculation is driven by high spatio-temporal resolution weather conditions simulated by the Weather Research and Forecasting (WRF) model. However, a rigorous evaluation of the accuracy of different trajectory models on simulated long-distance migration is lacking. Hence, in this study our trajectory model was evaluated by a migration event of the corn earworm moth, *Helicoverpa zea*, in Texas, USA on 20–22 March 1995. The results indicate that the simulated migration trajectories are in good agreement with occurrences of all pollen-marked male *H. zea* immigrants monitored in pheromone traps. Statistical comparisons in the present study suggest that our model performed better than the popularly-used HYSPLIT model in simulating migration trajectories of *H. zea*. This study also shows the importance of high-resolution atmospheric data and a full understanding of migration behaviors to the computational design of models that simulate migration trajectories of highly-flying insects.

## 1. Introduction

Migration is a widespread phenomenon in insects, especially seasonal long-distance transport of insects in prevailing airstreams hundreds of meters aloft to quickly and successfully exploit favorable habitats [1,2,3]. As roles of huge insect “bioflow” in ecosystem services, processes and functions were well realized [4,5,6], the studies of insect migration have been strongly required and progressively more essential. However, the high-altitude flight and relatively small body size of windborne insects suggest that it is difficult to directly observe or trace the entire migration process of windborne insects. Therefore, computer simulation of migration trajectories is the most suitable and convenient technique for modelling and predicting the comprehensive migration pathways, processes, sources and landing areas of windborne insects. So far, many methods developed for trajectory simulation have contributed greatly to our knowledge of insect migration [7].

Migration trajectories were calculated firstly on the desert locust, *Schistocerca gregaria*, by the simplest algorithm in a computer program named “Subroutine WIND”, which reproduced wind systems regardless of meteorological accuracy [8,9]. To identify possible sources and predict downwind displacement of planthoppers, 6-h synoptic charts were computed by applying a streamline-isotach method [10]. Westbrook et al. [11,12] estimated the flight displacement of corn earworm moths by computing the hourly wind velocity data at 500 m above ground level (AGL). Specifically, the forward wind trajectory was simply simulated with rawinsonde wind velocity data by linear interpolation, which was at 12-h time interval and obtained from US National Weather Service (NWS). Moreover, backward trajectories were produced to estimate the take-off location of radar-observed insects by employing the UK Met Office’s dispersion model, Numerical Atmospheric Modeling Environment (NAME) [13], which was developed using the Unified Model (UM) analyses of wind evolution in space and time to solve air attribution problems. These models were able to explain the dispersal trajectory of migratory insects, nevertheless: (1) the principle and calculation processes of these methods are relatively simple so, that it was hard to guarantee the accuracy of trajectory results; (2) calculation of the trajectory is strongly affected by the spatio-temporal resolution of original weather data, while wind data used in those models is merely at long-time intervals such as 6 or 12 h and with a 1° to 2.5° horizontal resolution; (3) wind vectors were simply linearly interpolated to a particular pressure level or altitude.

To improve analytical quality of trajectory models, the Fifth-Generation Penn State/National Center for Atmospheric Research (NCAR) Mesoscale Model (MM5) [14], which can increase the spatio-temporal resolution of wind vectors, was applied in conjunction with GEARN (a particle dispersion model, Three-dimensional Atmospheric Dispersion Model for synoptic scale) [15] to describe the detailed migration process of planthoppers [16], of which the calculated trajectories are smooth and well performed. Moreover, the Hybrid Single-Particle Lagrangian Integrated Trajectory (HYSPLIT) model of the National Oceanic and Atmospheric Administration (NOAA) [17], which is designed for computing three-dimensional trajectories of air parcels, was applied extensively to study migratory trajectories of many insect species, such as aphids, midges, moths and rice planthoppers [18,19,20,21]. Recently, Westbrook et al. [20] also used the EDAS (Eta Data Assimilation System) data archive (U.S., 2004–present, 40-km, 3-hourly) to run the HYSPLIT model for simulating the displacements of air parcel-like fall armyworm moths that took-off at 500 m AGL. These previous studies also found that higher spatio-temporal resolution weather simulation significantly improved accuracy of migration trajectory of insects.

Although the HYSPLIT model is very easy to use, the custom parameters are difficult to define. In contrast with air parcels, pollens, seeds and spores, insect migrants, especially nocturnally high-flying moths, play an active role in their windborne flights [3,22,23]. Strong evidence for the flight behaviors of windborne moths at high altitude were provided using radar networks [24,25]. Those high-flying moths have been demonstrated to perform common orientation close to the downwind direction that guide their migratory flight pathways in seasonally-favorable directions [3,26]. The flying airspeeds of about 5 m/s of nocturnally migrating moths aloft added to the wind vector considerably increase the migratory distance and facilitate rapid long-distance transport [3,13]. The adaptive evolution of these flight behaviors has allowed them to move in favorable directions and rapidly reach suitable habitats [3]. Thus, the trajectory simulation of insect long-distance migration would be inadequate without regard for self-powered migratory behavior of airborne insects.

A new numerical trajectory model was developed based on a three-dimensional trajectory analysis [7,27] program, which is a Fortran program project added the self-powered flight behavior parameters of the target insect (including departure time, airspeed, heading direction and flight duration) for the flight trajectory calculation of insect migrants [28] (China National Copyright of Computer Software No. 2015SR090706, 2015), and on high spatio-temporal resolution wind fields reproduced by a next-generation meso-scale numerical weather prediction system, Weather Research and Forecasting (WRF) model. Specifically, the WRF model is a more advanced version of MM5 and provides good performance because of a multiple relocatable nesting capability, improved physics and advanced numerical simulation techniques [29]. Our numerical trajectory model has been frequently used to investigate the possible fallout locations and to track the source areas of armyworms [30], rice leaf folders [31] and rice planthoppers in East Asia [32,33]. However, this new trajectory model has not yet been evaluated rigorously, and lacks comparison on accuracy with other modelling methods. This is also a common problem for other trajectory simulation methods, due to very little observation data showing the whole migration process of windborne insects.

**Figure 1:**
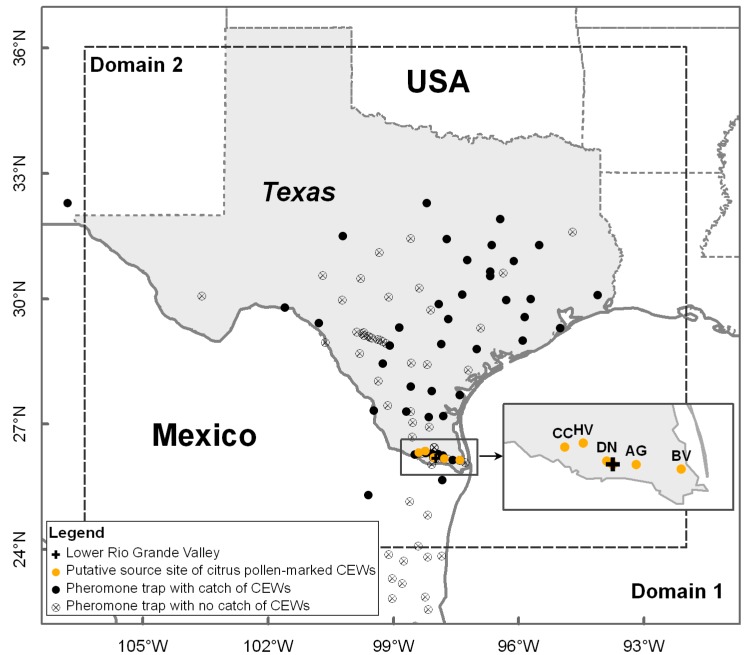
Locations of pheromone traps and occurrence of *H. zea* migrants (CEWs). Rectangle frame indicates locations in southern Texas with blooming citrus recorded in historical data and regarded as source sites in this study, comprising Adams Garden (AG), Bay View (BV), Citrus City (CC), Donna (DN) and Heald Valley (HV).

In the present study, a migration event of the corn earworm, *Helicoverpa zea* (Lepidoptera: Noctuidae) in Texas, USA on 20–22 March 1995 was selected to evaluate the performance of our model on migration trajectory simulation by comparisons with historical data and trajectories calculated by the popularly-used HYSPLIT model. The population dynamics and migration behaviors of high-flying *H. zea* migrants have been well investigated by a long-term application of radars at Lower Rio Grande Valley (LRGV, Figure 1) [23,34]. Significant progress on the whole migration path of *H. zea* population migration has been achieved since citrus pollen became a natural site of evidence for detecting an immigration event [35,36,37] during the citrus blooming period in February and March [38]. It was confirmed further by using tetroons and pheromone traps that citrus pollen-marked *H. zea* originate from the LRGV of northeastern Mexico and southeastern Texas, where populations of billions of *H. zea* occur [39,40]. Moreover, we would also assess the results of the numerical trajectory simulation model by comparisons with previous findings reported by Westbrook et al. [11].

## 2. Materials and Methods

### 2.1. Study Area and Occurrence of H. zea During 20–22 March 1995 in South-Central Texas

Corn earworm pheromone trap data in south-central Texas in spring 1995 is provided by USDA-ARS, College Station, Texas, USA. Male *H. zea* moths were checked daily from 20–22 March 1995 in a network of 109 pheromone traps [41] in Domain 1 (Figure 1), including 94 pheromone traps in Texas, USA, 1 in New Mexico, USA and 14 in Mexico. Additionally, *H. zea* males captured in 52 of these pheromone traps were frozen and examined for citrus pollen contamination on the body exterior by scanning electron microscope, according to the methods described by Bryant et al. [36]. The examination result of citrus pollen-marked *H. zea* migrants in pheromone traps was used to evaluate the new numerical trajectory model. Detailed occurrence data of pollen-marked *H. zea* during 20–22 March 1995 in south-central Texas are presented in Table 1. Pheromone traps were deployed to collect CEW moths at five locations (*viz* Bay View, Texas (BV, 97.40° W, 26.14° N), Donna, Texas (DN, 98.03° W, 26.21° N), Citrus City, Texas (CC, 98.39° W, 26.33° N), Adams Garden, Texas (AG, 97.78° W, 26.18° N) and Heald Valley, Texas (HV, 98.23° W, 26.36° N)) that spanned the major axis of the citrus production region of the LRGV region (Figure 1). There were numerous other citrus orchards in the LRGV, but the five reported trap locations (Figure 1) represent the extent of the citrus production region, and were in peak bloom at the time *H. zea* collections were made. Therefore, use of the five trajectory source locations represent samples within a confirmed source area of both CEWs and the unique citrus pollen (marker). Specifically, general occurrence of *H. zea* in blooming citrus groves at these 5 locations from 19–21 March 1995 (the previous day of sample days) was also revealed by the pheromone traps: BV (average daily number of trapped *H. zea* males was 2), DN (4), CC (8), AG (12) and HV (3).

### 2.2. Trajectory Modelling Methods

#### 2.2.1. HYSPLIT Model Simulation of *H. zea* Migration

The nocturnal airborne displacements of *H. zea* migrants taking off from the LRGV region were investigated by calculating forward air-mass trajectories using the HYSPLIT model, in which *H. zea* were assumed to be air particles. *H. zea* migrants typically initiate migratory flight at 30 min after sunset (19:00 h local time (same thereafter) in late March 1995) and fly at around 500 m above ground level (AGL) for a flight duration of up to 12 h [34,42,43]. However, they are likely to land at any time. Based on these observations, a 12-h forward trajectory starting at a given source area was calculated at 19:00 h the local time for each day during 20–22 March 1995 in HYSPLIT model. Additionally, starting heights were set at 500, 550, 600, 650 and 700 m AGL of all source sites [34,43]. Thus, the simulations were based on the wind data obtained from the National Center for Atmospheric Research/National Centers for Environmental Prediction (NCAR/NCEP) North American Regional Reanalysis (NARR) products at 29 pressure levels. The initial NARR data is 3-hourly and with a spatial resolution of 32 km (approximate 0.33°). The simulated output from HYSPLIT was with a temporal resolution of 1-h.

#### 2.2.2. WRF Model Simulation of *H. zea* Migration

The WRF Model (www.wrf-model.org) [44] in this study was applied by using a two-way nesting approach to drive the programming of three-dimensional trajectory analysis. The WRF model is designed for both atmospheric research and operational forecasting needs. In addition, it is a fully compressible, nonhydrostatic model and its capability of two-way nesting can well produce the regions of interest with extremely fine grid resolutions. The model outputs can reveal atmospheric features influencing the dynamic migration process, and be deployed to construct the forward trajectories by supplying initial and boundary conditions with a time series of simulated temperature, wind speed, wind direction, and precipitation for locations on a horizontal grid at vertically-spaced sigma levels. Figure 1 illustrates the calculated domains and detailed model setup and parameterizations are listed in Table 2. Additionally, the NARR data products were used as inputs to drive the WRF model. The final simulated output from WRF in the studied domain was at an hourly time-step and a 5.667-km horizontal resolution.

For comparisons of simulation quality between different modelling methods, we ran a forward trajectory analysis program of inert particles run under UBUNTU 14.04 (https://www.ubuntu.com/index_kylin) (named ‘WRF0 model’ in this study), assuming the *H. zea* migrants were inert particles passively transported by the wind. To present the comparison with the normal trajectory modeled by HYSPLIT, the 12-h forward trajectories departing from 5 source areas were all calculated hourly at the same start time (19:00 h the local time) with the same initial heights (500, 550, 600, 650, 700 m AGL).

For evaluation of the effects of flight behaviors on trajectory distance and migration direction, a single-night flight of emigrating *H. zea* from the LRGV and the likely source areas of pollen-marked *H. zea* males caught in pheromone traps in south-central Texas were modeled separately by another forward trajectory analysis program run under UBUNTU 14.04. When running the migratory *H. zea* trajectory analysis program (named “WRF1 model” in this study), we assumed that (1) *H. zea* migrates downwind and is given a constant air speed of 4.5 m/s [23]; (2) *H. zea* migrants exhibit common orientation to the right of the downwind vector, and the crab angle (difference between insect alignment and wind displacement direction) is 30° [22,23]. By adding these simple flight behaviors to the local airflow simulated from the WRF model, the flight trajectory of migratory *H. zea* was calculated. The 12-h forward transports of *H. zea* were calculated in WRF1 model at the same altitudes, starting time and source areas as that designed in HYSPLIT and WRF0 models.

The endpoints data and migration trajectories were displayed with ArcGIS 10.0 (EISR Inc., Redlands, CA, USA), while transporting wind fields also derived from NARR data products were displayed with Grid Analysis Display System (GrADS) Version 2.0.1 (COLA, Fairfax, VA, USA; http://cola.gmu.edu/grads).

### 2.3. Evaluation of the Modelling Methods

Three modelling methods described above were evaluated by checking whether all the pheromone traps with citrus pollen-marked *H. zea* adults captured were located in the sweeping area of all forward trajectories of each model. The assessment of simulation accuracy was based on assessing the agreement by examining the spatial relationship between model outputs and observations for each sampling date (i.e., the occurrence of pheromone trap catches of pollen-marked *H. zea* migrants in Texas during 20–22 March 1995). The spatial relationship between the simulated endpoints and the geographic positions of pheromone traps with pollen-marked *H. zea* adults, which are typically represented by straight-line distance (Euclidian distance) between points [45], were explicitly measured by utilizing the Point Distance Tool in ArcGIS 10. 0 (EISR Inc., Redlands, CA, USA) [46]. As *H. zea* moths can land at any time during their flight movement, the shortest linear distances from each pollen-marked trap to simulated flight pathways separately obtained by the HYSPLIT and WRF0 models of inert particles (i.e., both are strictly atmospheric models) and WRF1 simulations of *H. zea* migrants (i.e., WRF1 model expands upon WRF0 by incorporating insect migratory flight behaviors), were computed, named SDP_toH_, SDP_toWRF0_ and SDP_toWRF1_, respectively. Further, the linear distances between each pollen-marked trap and the nearest simulated hourly endpoints of the HYSPLIT, WRF0 and WRF1 trajectories were calculated as PtoP_H_, PtoP_WRF0_ and PtoP_WRF1_, respectively. We evaluated output accuracy by comparing these linear distances (each treatment method: *n* = 14) among the three modelling methods using Wilcoxon signed-rank test in JMP 13.2.0 software (SAS Institute Inc., Cary, NC, USA) [47]. Meanwhile, calculated trajectories and landing locations from HYSPLIT and WRF0 models were compared with each other and with findings concluded by Westbrook et al. [12] to illustrate further evaluation.

The trajectory distances of *H. zea* migrants emigrating from their origins were obtained by calculating the sum of straight-line distances between endpoints in time series (at two consistent model time steps (each point is at 1 h)). Specifically, each of the 75 trajectories (5 start locations × 1 start time × 5 start heights × 3 nights) were simulated from the HYSPLIT, the WRF0 and the WRF1, respectively.

The degree of drift angles (the direction of migration trajectory with and without flight behaviors simulated by the numerical modelling method drifted from the standard HYSPLIT trajectory, named DAH_WRF0_ and DAH_WRF1_, respectively) of each paired-groups (i.e., WRF0 model vs WRF1 model) were calculated at each 4 h (i.e., migration direction calculated at flight durations of 4, 8 and 12 h after departure at 19:00 h local time, named D4h, D8h and D12h, respectively). We compared the differences of migration directions (i.e., DAH_WRF0_ and DAH_WRF1_, each method: *n* = 225) and trajectory distances obtained by the HYSPLIT, WRF0 and WRF1 models (each method: *n* = 75) using the Wilcoxon signed-rank test. All *p* values ≤ 0.05 were considered statistically significant.

## 3. Results

### 3.1. Long-Distance Migration Processes of Pollen-Marked H. zea Males Over Texas on 20–22 March 1995

**Figure 2:**
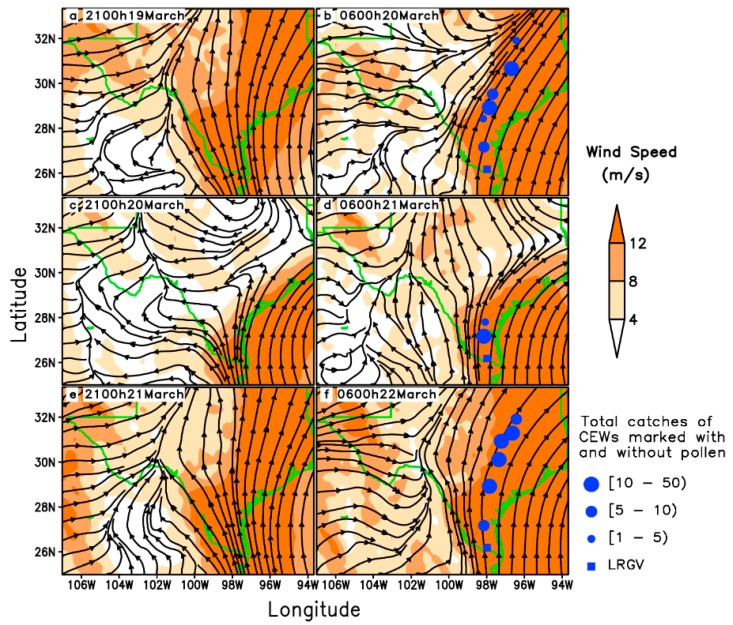
Wind fields at 950 hPa from 21:00 h to 06:00 h the next morning (local time) and pheromone trap catches of *H. zea* (CEWs) in Texas on 19–21 March 1995. Streamlines present wind fields; solid circles indicate locations where citrus pollen-marked CEW was captured.

**Figure 3:**
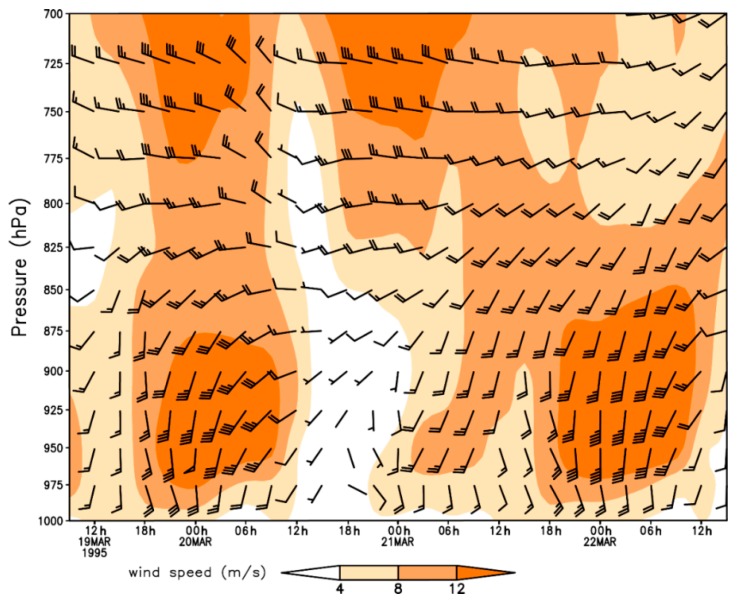
Vertical profile wind field with pressure-altitude and time for the period of 19–21 March 1995 at Karnes City, Texas, USA.

Nocturnal long-distance migration processes of *H. zea* on 20–22 March 1995 in Texas were reconstructed based on detections of citrus pollen on the body of trapped *H. zea* (Table 1). The pollen-marked *H. zea* were captured most frequently at Falfurrias (latitude: 27.17° N, 105 km from source areas) on all sampling days, and the northernmost location trapping pollen-marked *H. zea* males was Richland, Texas (latitude: 31.91° N, 637 km from source areas), which corroborated the well-established long-distance migration of *H. zea* population. The southerly winds at 950 hPa (approximately 550 m above ground level) predominated during the nights on 19–21 March 1995 in eastern Texas (Figure 2), while westerly wind with low wind speed predominated in western Texas. Under such situations, *H. zea* males emigrating from the given source areas were carried northward. The southerly low-level jets (LLJs, wind speeds > 12 m/s) during these study-period nights compared well with the distributions of pollen-marked *H. zea* males. Specifically, the fast-southerly winds prevailed over eastern Texas with wind speed exceeding 12 m/s (Figure 2a,b), and westerly airflow prevailed over western Texas on the night of 19 March when there was a distinctive wind shear line along 102° W longitude at 21:00 h (Figure 2a). On the morning of 20 march, the westerly winds strengthened, while the southerly wind veered and a southwesterly air current prevailed over central Texas (Figure 2b). During the night of 20 March, the southerly wind was weakened by the strong northerly and westerly winds from 21:00 h to 06:00 h the next day (Figure 2c,d). On the third night, the southerly wind accelerated to speeds greater than 12 m/s and predominated over eastern Texas (Figure 2e,f). It is noted that there were 2, 0 and 2 citrus pollen-marked *H. zea* males collected respectively on 20–22 March 1995 at Karnes City, Texas, which was more than 300 km from the LRGV. Southerly wind from 900–1000 hPa prevailed on the nights on 20 March and 22 March with beneficial conditions for northward movement of *H. zea* migrants, whereas the wind on 21 March was of variable direction and strong wind speed (Figure 3). Specifically, LLJs formed within 975–900 hPa and occurred from approximately 19:00 h (local time) on 19 and 21 March to 07:00 h the following day. The maximum wind speeds of these jets occurring at 925–950 hPa were intense (18–24 m/s). However, because of the light southerly transporting wind and moderate self-powered flight capability, no citrus pollen-marked *H. zea* males were trapped at Karnes City and its more northern areas on the night of 21 March. It is concluded that the immigration of *H. zea* males in Texas was coincident with the emergence of southerly LLJs in late March.

### 3.2. Statistical Comparisons of Simulations on H. zea Migration Trajectories over Texas

**Figure 4:**
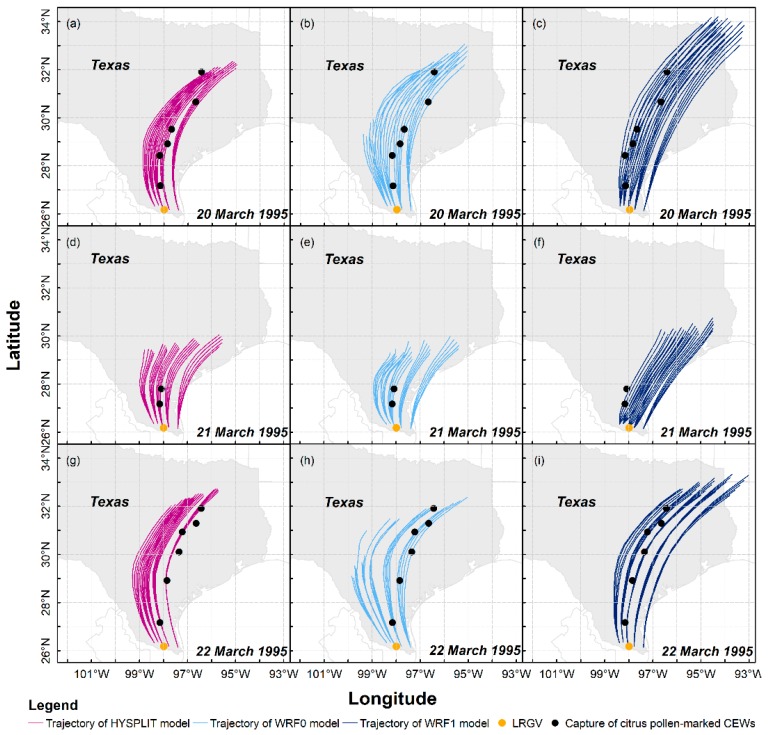
Migration trajectories of *H. zea* migrants (CEWs) simulated by three trajectory analysis models. The starting points of each trajectory, i.e., source sites, are listed in Figure 1.

As outcomes of wind fields displayed in Figure 2, 225 long-distance flight trajectories of *H. zea* migrants on 20–22 March 1995 were calculated using three trajectory modelling schemes, that is, HYSPLIT, WRF0 with no flight behavior of *H. zea* and WRF1 with the flight behavior (Figure 4), and 75 trajectories were obtained in each scheme, and all trajectories simulated from different modeling schemes present similar migration pattern of *H. zea* in Texas. Specifically, the synoptic background during the first computed night determined that the calculated trajectories extended northward in southern Texas first, then drifted northeastward over central Texas and the border of Texas and Louisiana (Figure 4a–c). The low wind speed at 950 hPa on the night of 20 March over Texas accounted for the short-distance dispersal of *H. zea* migrants departing from southern Texas (Figure 4d–f), and only Falfurrias and Alice, Texas (approximately 180 km from the LRGV) collected one pollen-contaminated *H. zea* male. The calculated trajectories on 22 March 1995 (Figure 4g–i) showed similar patterns with those on 20 March 1995 (Figure 4a–c). Due to westerly air currents over western Texas that were unfavorable for long-distance northward flights of windborne *H. zea* migrants, there were 5 and 11 trajectories calculated from WRF0 on the first and third night, respectively, tending to stretch westward but terminated before *H. zea* migrants completed the 12-h migration process.

Furthermore, 3 of 14 pheromone traps with pollen-marked *H. zea* caught were out of the sweeping areas on 20–22 March 1995 obtained from HYSPLIT (Figure 4a,d,g), while the location of Alice was slightly out of the sweeping area on 21 March 1995 calculated by using WRF1 (Figure 4f) and Richland was very close to the end of trajectories on 22 March 1995 of WRF0 (Figure 4h). Based on this evaluation, migration routes estimated with WRF0 and WRF1 performed better than those of HYSPLIT. Although these results indicate the high consistency of migration trajectory patterns, we intensively compared the migration trajectories of *H. zea* migrants simulated by three trajectory analysis models. Our results indicated that linear distances between pollen-marked moth capture points to the nearest (of 12-h) simulated migration trajectories, including SDP_toH_, SDP_toWRF0_ and SDP_toWRF1_, were 7.1 ± 9.4 km, 4.0 ± 3.1 km and 3.2 ± 3.7 km, respectively. Additionally, the mean linear distances from traps that had collected pollen-marked *H. zea* to the nearest (of 12 hourly) trajectory waypoint were 19.3 ± 16.9 km, 19.2 ± 19.1 km and 10.1 ± 4.0 km for PtoP_H_, PtoP_WRF0_ and PtoP_WRF1_. Both comparison tests of SDP and PtoP showed that all simulated trajectories are consistent with the actual pollen-marked migration paths, and the transport of inert particles derived from the HYSPLIT and the WRF0 models were not different (Wilcoxon signed-rank test: *p* > 0.1). The results from these two models consistently indicated that the pollen-marked *H. zea* males (actually, air particles) trapped on 20 March 1995 in Rockdale (latitude: 30.66° N, 507 km from source areas) were tracked back to BV of southern Texas. On the following day, DN was suggested to be the source area for pollen-marked *H. zea* adults collected in Falfurrias. On 22 March 1995, part of the emigratory population from BV most likely terminated their long-distance migrations when they reached Rogers (latitude: 30.93° N, 527 km from source areas), while some *H. zea* migrants departing from AG landed in Falfurrias. Moreover, the nearest hourly endpoints of *H. zea* reproduced from the WRF1 model on 20–22 March 1995 were significantly closer to pheromone traps collecting pollen-marked *H. zea* males than inert particle dispersal pathways calculated from the HYSPLIT and WRF0 (one-tailed Wilcoxon signed-rank test: *p* = 0.0389 and *p* = 0.032, respectively). Additionally, the capture of pollen-marked *H. zea* males in Richland on 20 March 1995 could not be explained by the HYSPLIT model simulation but could be explained by the WRF0 and WRF1 model simulations. This result demonstrated that a spatial resolution of 5.67 km higher than 32 km employed in trajectory model showed a better performance in simulation of migration trajectory, and the WRF1 model had more accuracy on simulating the migration trajectory of *H. zea* moths than the HYSPLIT and WRF0 models for inert particles.

**Figure 5:**
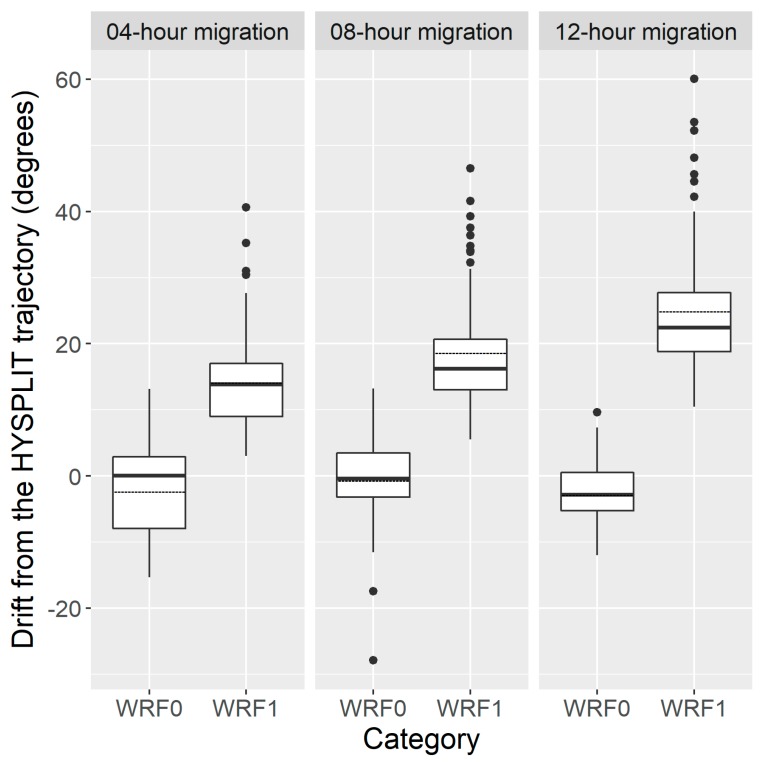
The degrees of trajectory direction of *H. zea* migration trajectories calculated by WRF0 and WRF1 drifting from the standard Hybrid Single-Particle Lagrangian Integrated Trajectory (HYSPLIT) trajectory of three flight durations, respectively. The bottom and top of the box indicate the lower and upper quartile values, respectively. The horizonal solid black line shows the median for each category, and the black dashed line represents the mean. Whiskers indicate the 5th and 95th percentiles, while the black circle represents the outlier.

The mean drift directions of 4-h, 8-h and 12-h migration trajectory of inert particles and *H. zea* migrants were significantly different (Figure 5) (for the flight duration of 4 h (FD4h): mean (±1 standard deviation (SD)) DAH_WRF0_ = −2.2 ± 6.7°, DAH_WRF1_ = 25.1 ± 10.1°; *p* < 0.0001; for FD8h: mean (±1 SD) DAH_WRF0_ = −2.7 ± 4.7°, DAH_WRF1_ = 18.8 ± 8.9°; *p* < 0.0001; for FD12h: mean (±1 SD) DAH_WRF0_ = −0.5 ± 6.6°, DAH_WRF1_ = 14.3 ± 7.6°; *p* < 0.0001). Thus, migration trajectories simulated using the WRF1 model and WRF0 model presented a similar migration pattern, but had 19.4 ± 9.9° and −1.9 ± 6.2° drift when compared with those calculated with the HYSPLIT, respectively. For instance, the results showed that source area of *H. zea* immigrants in Rockdale on 20 March 1995 was determined to be BV by both inert-particle trajectory models, while the WRF1 model indicated that Rockdale immigrants probably originated from HV. On 21 March 1995, pollen-marked *H. zea* males captured in Falfurrias were estimated to have come from CC using the WRF1 model, rather than from DN as calculated by the HYSPLIT and WRF0 models. On the third day, the result of the WRF1 model suggested that DN and HV provided the source population of *H. zea* that migrated to Falfurrias and Rogers, which is different from the outcomes computed by the HYSPLIT and WRF0 models.

**Figure 6:**
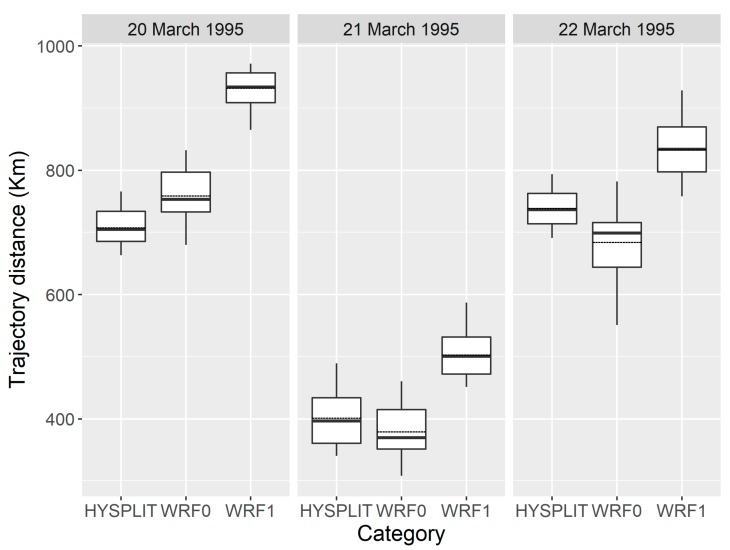
The distance from the starting source area to the endpoints of *H. zea* migration trajectories calculated by the HYSPLIT, WRF0 and WRF1, respectively. The bottom and top of the box indicate the lower and upper quartile values, respectively. The horizonal solid black line shows the median for each category, and the black dashed line represents the mean. Whiskers indicate the 5th and 95th percentiles.

Furthermore, the transport distance of inert particles calculated by the WRF0 model agreed well with that simulated using the HYSPLIT model (Figure 6) (*p* = 0.5209), but significantly differed from the flight distance of *H. zea* migrants (*p* < 0.0001). Specifically, *H. zea* migrants traveled an additional 146.8 ± 36.4 km during each 12-h trajectory simulation at each sampling night, which was significantly further than inert particles (one-tailed Wilcoxon signed-rank test *p* < 0.0001). Incorporating common orientation and self-powered airspeed of insect migrants to the modelling method considerably improved the simulation of migration trajectories.

## 4. Discussion

In this study, the nocturnal immigration process of *H. zea* migrants during 20–22 March 1995 was well explained, and the simulated migration trajectories were reproduced by the HYSPLIT and numerical trajectory modelling methods. The numerical trajectory model correctly represented the windborne transport of inert particles, and these trajectories showed similar patterns with two single trajectories at 500 m AGL in a case study of the immigration events during 18–24 March 1995 described by Westbrook et al. [12]. This is because flight pathways of *H. zea* were greatly associated with the evolution of transporting wind and wind patterns on the nights studied [11,41], which was also consistent with the synoptic conditions reported by Westbrook et al. [12]. Specifically, wind-assisted transport of *H. zea* migrants from source areas in southern Texas was directed by strong southerly and southeasterly winds, especially the prevailing LLJs that contributed to *H. zea* migrants’ successful long-distance migration [12,43]. Although it is still unclear how high-flying insects select the most favorable and fastest winds [3] the spatio-temporal distributions of the LLJs acting as the “conveyor belt” [48] coincided with the immigration events of *H. zea* in Texas, aphids [18,49], rice planthoppers [50,51], and other long-distance transportation moths [48,52].

Our statistical and trajectory analyses suggested that the WRF0-calculated trajectories of inert particles on 20–22 March 1995 well matched the outputs from the HYSPLIT model. To consider the model quality on *H. zea* migration trajectory, the shortest distances of both simulated trajectories and hourly endpoints from each pheromone trap collected with pollen-marked *H. zea* migrants were compared between different treatment groups. Our results showed that the numerical trajectory model incorporating flight behaviors of migratory *H. zea* (i.e., WRF1 model) had the best performance, of which the average shortest distances were significantly smallest. This can be achieved because a re-analysis of wind fields with a high spatial resolution of 5.67 km using the WRF model was more accurate than the original weather dataset of 32 km employed in the HYSPLIT model. Moreover, larger occurrence of trapped *H. zea* moths in citrus-blooming groves of the western LRGV region represented the possibility of a relatively high number of *H. zea* emigrating. This is consistent with the fact that the geographical positions of all pheromone traps with pollen-marked moths captured formed a line and were originated from the western part of the LRGV region, which was well explained by trajectories on 20 and 22 March 1995 simulated by using WRF1. It is concluded that the numerical trajectory model in the present study can accurately hourly-track the possible landing locations of high-flying insects, which will increase the understanding of long-distance migration dynamics of the highly mobile insect population between regions and between years.

The statistical comparisons of migration distance and dispersal direction between each paired group illustrated that the flight trajectories of windborne *H. zea* stretched more northward (146.8 ± 36.4 km further than passively-transported inert particle) and had a slightly eastward drift (19.4 ± 9.9° to right of displacement path of inert particle). The results suggest that the addition of migration behaviors to the trajectory model had a considerable impact on the migration path of *H. zea* moths. This conclusion was also supported by Wood [53] that an additional airspeed of high-flying insect to wind speed greatly increased the transport distance, in detail, *H. zea* migrants could travel a further 63 km in 5 h than the dispersal distance of the air parcel. It was observed by vertical-looking entomological radar that the flight behavior facilitated rapid long-distance movements of moths, which were transported an extra 97.8 ± 5.4 km within each 8-h simulation [3]. Furthermore, during this *H. zea* migration event when the crab angle was considered, the flight pathways were significantly to the right of air parcel dispersal and stretched more eastward. It is shown that the flight headings of highly-flying insects, including *H. zea* migrants, would induce their lateral displacements and have a substantial effect on their migration pathways [3,26]. Nevertheless, simply regarding wind vector as an individual’s displacement in previous studies [11,12,20] might increase the error of simulations for the transport of “medium-sized” (10–70 mg) or larger moths [26] capable of self-powered flight behaviors [3]. It is suggested that more emphases should be put on quality of meteorological fields and the knowledge of flight behaviors of insect migrants.

Under clear and calm weather on 19–22 March 1995, pollen-marked *H. zea* males were indicated to terminate the flights at any time and somewhere, but uniformly in the direction of the prevailing wind and along their migration routes (Figure 2, Table 1). It is suggested by previous studies that some migratory insects cease to fly because of encounter with favorable habitats [54,55] or their limited flight capacity, which exhibits great differences among individuals [56]. The presence of pollen-marked *H. zea* males in Texas was occasional, however, the pheromone traps in Texas were checked daily, so that it was difficult to decide when they landed. By contrast, the taking-off time of *H. zea* had been well studied [34,43]. Thus, in this case study the backward trajectory simulation designed to identify source areas would have less capability than the forward simulation, due to the absence of high-frequency observation data. In addition to their moderate flight capability or intrinsic landing behavior, high-flying insect migrants, including *H. zea* moths, can be forced to descend by unfavorable atmospheric factors or phenomena, particularly by low temperature and rainfall [48,57]. Because it is assumed that *H. zea* generally do not fly at air temperatures less than 10 °C [11]; it is noted that, throughout the study period, the air temperature at 950 hPa in Texas was much greater than 10 °C for the flight of *H. zea* and there was no rain in the study region. The weather on 19–22 March 1995 remained favorable for *H. zea* already in flight [12], and no migration trajectories were forced to stop because of low air temperature or precipitation. Although both the low temperature threshold and hourly precipitation for insect flight were not considered in this case study, meteorological limitations had been set in the trajectory analysis program to stop the calculation of *H. zea* migration trajectories. Flight behavior parameters can be easily edited according to other studied insect species. Also, the trajectory model will stop calculation of migration trajectories that extend beyond the simulated domain.

While our results are in good agreement with pheromone trap studies and the three-dimensional trajectory model presented is flexibly adjusted, the behavioral adaptations of most insects undertaking long-distance migrations remain largely unknown. For instance, propensity for multiple-night flight of *H. zea* is still poorly understood [43,58]. As presented in this study, pollen-marked *H. zea* traveled more than 600 km in one night (9–10 h), reaching at Richland, Texas with the assistance of a LLJ. All immigration events on 20–22 March 1995 were well analyzed by forward trajectory simulations. However, if monitoring data for other immigration events are absent for a specific time or location or if the number of flight nights are unknown, a dramatic error in predicting the migration pathways would appear. Hence, successive emigration capability of *H. zea* remains to be elucidated to improve the accuracy in predicting long-distance migration [43,58]. Although little is known about re-migration of *H. zea* and whether capture of pollen-marked moths in pheromone traps represent the end points of migration over multiple nights rather than the single one, pollen may be a transient marker especially when the moths also feed on other host plants [59]. Additionally, neither northerly wind nor low wind speeds at previous nights during 16–18 March 1995 (Figure S1) were favorable for long-distance, northward migration of *H. zea* moths into northern Texas. Thus, it can be concluded that the captures of citrus pollen-marked *H. zea* moths in northern and central Texas represented the endpoints of migration over less than two nights in this study. Even if the moths arrived on the previous day, linear distance between Alice and the trajectories of WRF1 taking place at the night of 20 March 1995 was only at 960 m. It reinforced that in this study WRF1 has the best performance for tracking a corn earworm moth migration pathway. The present study is a case study aimed at comparing the performance of numerical trajectory simulation methods, but more work is still needed to improve the prediction of migratory insects of public interest.

## 5. Conclusions

A new numerical trajectory model based on the Weather Research and Forecasting (WRF) model and incorporating insect flight behavior parameters improved performance in simulating insect migration. Specifically, the forward trajectories calculated from this new model were well agreed with the occurrence of pollen-marked *H. zea* males during 20–22 March 1995 in Texas, USA. Statistical comparisons in the present study suggest that our model has a better performance than the popularly-used HYSPLIT model in simulating migration trajectories of *H. zea*. The approach framework of the present study is able to be widely adopted in simulating trajectories of migratory insects of public interest.

## Figures and Tables

**Table 1:** Pheromone trap catches of citrus pollen-marked *H. zea* males on 20–22 March 1995 in Texas, USA.

Date	Location	Position	Number of Trap Catches	Number of *H. zea* Examined for Pollen	Number (Percentage (%)) of *H. zea* with Citrus Pollen
20 March	Falfurrias	98.15° W, 27.17° N	6	4	1 (25%)
	Three Rivers	98.18° W, 28.43° N	2	2	1 (50%)
	Karnes City	97.85° W, 28.92° N	19	2	2 (100%)
	Belmont	97.68° W, 29.52° N	9	3	2 (66.67%)
	Rockdale	96.68° W, 30.66° N	11	1	1 (100%)
	Richland	96.43° W, 31.91° N	3	3	3 (100%)
21 March	Falfurrias	98.15° W, 27.17° N	10	2	2 (100%)
	Alice	98.08° W, 27.80° N	2	1	1 (100%)
22 March	Falfurrias	98.15° W, 27.17° N	8	4	1 (25%)
	Bastrop	97.35° W, 30.11° N	14	2	1 (50%)
	Kosse	96.64° W, 31.29° N	14	2	1 (50%)
	Richland	96.43° W, 31.91° N	8	3	3 (100%)
	Rogers	97.22° W, 30.93° N	21	5	1 (20%)
	Karnes City	97.85° W, 28.92° N	21	2	1 (50%)

**Table 2:** Selection of scheme and parameters of the Weather Research and Forecasting (WRF) model.

Item	Domain 1	Domain 2
location	31.16° N, 99.21° W	31.16° N, 99.21° W
the number of grid points	100 × 96	256 × 232
distance between grid points (km)	17	5.667
layers	45	45
map projection	lambert	lambert
microphysics scheme	WSM3	WSM3
longwave radiation scheme	RRTM	RRTM
shortwave radiation scheme	Dudhia	Dudhia
surface layer scheme	Monin-Obukhov	Monin-Obukhov
land/water surface scheme	Noah	Noah
planetary boundary layer scheme	YSU	YSU
cumulus parameterization	Kain-Fritsch (new Eta)	Kain-Fritsch (new Eta)
simulated time	every 24 h	every 24 h

## Supplementary Materials

The following are available online at http://www.mdpi.com/2075-4450/9/3/115/s1, Figure S1: Mean wind field at the night of 16–18 March 1995, respectively.
