# Supplementary figures and images for: An Advanced Numerical Trajectory Model Tracks a Corn Earworm Moth Migration Event in Texas, USA
Source: Insects. 2018 Sep 5;9(3):115. doi: 10.3390/insects9030115 (PMC6163387; doi:10.3390/insects9030115)

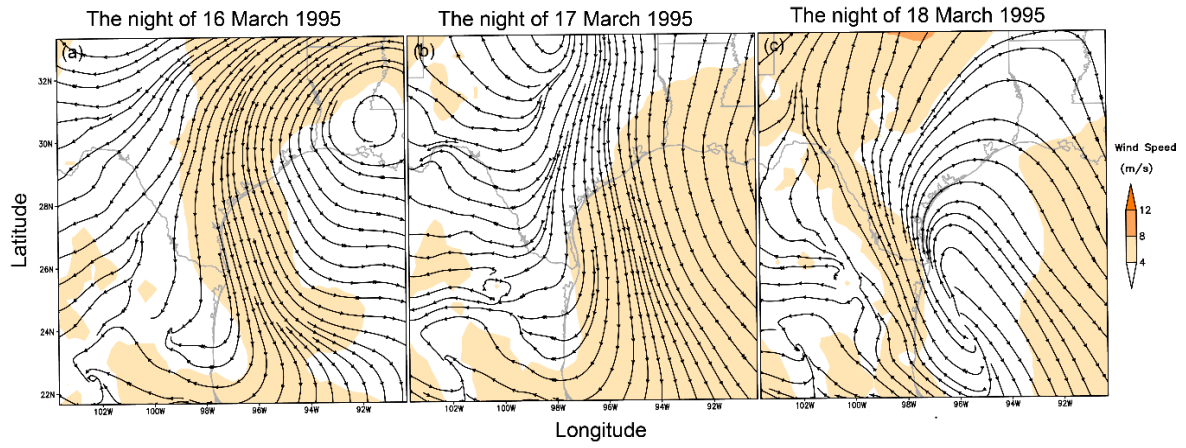

**Figure S1.** Mean wind field at the night of 16–18 March 1995, respectively. Streamlines present wind fields.

Supplement: Supplementary file 1 [file insects-09-00115-s001.pdf]
